# A Qualitative Analysis of the Needs and Experiences of Hospital-based Clinicians when Accessing Medical Imaging

**DOI:** 10.1007/s10278-021-00446-1

**Published:** 2021-04-08

**Authors:** Séan Cronin, Bridget Kane, Gavin Doherty

**Affiliations:** 1 Trinity College Dublin, Dublin, Ireland; 2 Karlstad University, Karlstad, Sweden

**Keywords:** PACS, Digital imaging, Sterile, Infection control, User–computer interface, Touchless, Gestures, Hands-free

## Abstract

As digital imaging is now a common and essential tool in the clinical workflow, it is important to understand the experiences of clinicians with medical imaging systems in order to guide future development. The objective of this paper was to explore health professionals’ experiences, practices and preferences when using Picture Archiving and Communications Systems (PACS), to identify shortcomings in the existing technology and inform future developments. Semi-structured interviews are reported with 35 hospital-based healthcare professionals (3 interns, 11 senior health officers, 6 specialist registrars, 6 consultants, 2 clinical specialists, 5 radiographers, 1 sonographer, 1 radiation safety officer). Data collection took place between February 2019 and December 2020 and all data are analyzed thematically. A majority of clinicians report using PACS frequently (6+ times per day), both through dedicated PACS workstations, and through general-purpose desktop computers. Most clinicians report using basic features of PACS to view imaging and reports, and also to compare current with previous imaging, noting that they rarely use more advanced features, such as measuring. Usability is seen as a problem, including issues related to data privacy. More sustained training would help clinicians gain more value from PACS, particularly less experienced users. While the majority of clinicians report being unconcerned about sterility when accessing digital imaging, clinicians were open to the possibility of touchless operation using voice, and the ability to execute multiple commands with a single voice command would be welcomed.

## Introduction

PACS has become a key part of the digital hospital workflow, alongside radiology information systems (RIS), dictation systems, preliminary report systems, and electronic health records (EHR) [1]. Technology improvements that support clinical excellence and service to the community are more likely to be accepted by clinicians [2], and high levels of satisfaction with PACS were reported following its introduction [3].

Increasingly, hospital PACS have been integrated with a variety of EHR/EMR systems [4], including national systems such as the National Image Management Information System (NIMIS) in Ireland, which supports distributed access to medical imaging. While these integrated solutions bring many benefits, such integration requires adherence to industry standards in order to allow various systems to effectively communicate with each other. Despite these standards, there remain considerable challenges in the use of these systems. Furthermore, trade-offs are often made between the potential to collaborate and share images vs. the response time and image resolution.

Now that digital imaging has become embedded in clinical practice, it is timely to examine clinician experiences with PACS, in order to inform future development of these systems. Recent technological developments have opened possibilities for a wider range of user interaction modalities for operating computer systems; voice- and gesture-based interactions in particular have been proposed as having applications in interacting with digital imaging, particularly in surgery and interventional radiology [5], and so attitudes towards touchless interaction with digital imaging are also explored.

This paper addresses key questions regarding PACS implementation, and explores the potential for future development of the user interface of these systems:

1. How do health professionals (HP) use PACS?
2. What challenges do HPs encounter when using PACS?
3. What are HP attitudes to touchless interaction with PACS?

## Background

In this section, we provide a brief overview of previous relevant work on the adoption and usage of PACS.

### Adoption of PACS

The introduction of Picture Archiving and Communication Systems (PACS) to hospital environments has had a significant, positive impact on clinicians’ workflows. Lederman et al. observe that PACS has workflow influences beyond automation, having impact also on radiologist productivity, technologist productivity and clerical staff productivity, report-turnaround times, and communication between clinician and radiologist [2]. Specifically, PACS has increased the amount of useful information available to clinicians, and improved the availability of images, image quality, and the quality of patient care [6].

When implementing PACS, there are a number of factors that should be considered. Hospital-specific resources, capabilities and the use of PACS are strongly inter-related, and the involvement of multidisciplinary teams consisting of physicians, technicians, and engineer is a critical success factor when applying improvements to PACS [7]. The extent of PACS integration will impact the level at which it is used, as well as how it is incorporated into clinical practice, which is essential to realizing the potential benefits of PACS [8][9].

### Benefits of PACS

The literature argues for the benefits of a digital workflow incorporating PACS, and digital workflows are seen as inevitable [1]. It has been argued that the most significant of these benefits has been the ability to reengineer the overall workflow, allowing for the reduction of many inefficiencies that existed in diagnostic imaging departments [9]. Srinivasan et al. reported that the introduction of a digital radiology system improved clinician satisfaction and workflow, increased clinician image viewing [10].

Chen et al. noted that institutions without PACS were at a competitive disadvantage [12], particularly as PACS is considered sufficiently affordable to be within the reach of smaller institutions and more cost-conscious groups. This “commoditization of PACS” has resulted in decreased cost, improved interoperability, easier replacement, increased customer choice, and improved vendors’ response to their customers [12].

### Problems with PACS

Since the introduction of PACS, clinicians have become accustomed to reading radiology reports [13], resulting a significant reduction in the amount of radiologist/clinician face-to-face time [10, 11].

Another potential issue is the shift in expectations for radiology departments, with the diminished report turnaround time leading clinicians to increasingly expect immediate access to these reports [11]. On a related note, “when the urgency of a study is not appropriately managed, patient care decisions may be unnecessarily delayed, with possible adverse outcomes” [1].

While the initial rollout of PACS motivated a range of studies on efficiency and acceptability, now that these technologies are well established, it is opportune to examine the usage and experiences of health professionals, and problems experienced in practice.

## Methods

In order to investigate the experiences of health professionals with PACS, a qualitative approach based on semi-structured interviews was used. Interviews were conducted with clinicians in a range of roles, between February 27, 2019, and December 9, 2020. Research ethics approval was obtained from the relevant institutional ethics review committee.

### Sampling, Recruitment, and Consent

Participants were contacted using a combination of personal and professional contacts, as well as a hospital visit. The inclusion criteria were that participants were healthcare professionals and had experience of using PACS as part of their normal duties. Participants representing a range of perspectives, and participants with a high level of PACS usage were sought through purposive sampling. The sample size was deemed sufficient once saturation was reached.

Potential participants were contacted directly and were provided with a short summary of the goals of the study to gauge interest and relevance. Those who consented were given the choice between face-to-face interviews and over-the-phone interviews. Snowball sampling was used to recruit further participants. All individuals that expressed an interest in taking part in the study were provided with a time, date, and (in the case of face-to-interviews) a location for their interview. They were also provided with a participant information sheet and a consent form. No form of incentive was provided and no contacted individuals declined to take part.

### Participants

Thirty-five participants from five countries were interviewed (Ireland, UK, UAE, USA, and Australia) with interviewees being predominantly from Ireland. The sample included clinicians of various levels of seniority, and a range of roles. All interviewees were hospital-based healthcare professionals. Role, speciality, and experience for participants are provided in Tables 1 and 2.

**Table 1:** Participant characteristics

Role	Number of participants	Speciality	Years of experience with PACS
Intern	3	3 General	3 = 0-4
Senior House Officer/Resident	11	6 General	10 = 0-4
		1 Orthopedics	1 = 5-9
		1 Pediatrics	
		1Obstetrics & Gynecology	
		1 Anesthetics	
		1 Surgical	
Specialist Registrar/Fellow	6	2 Colorectal	1 = 5-9
		1 Orthopedics	5 = 10-14
		1 Neurosurgery	
		1 Pain	
		1 Unspecified	
Consultant/Attending	6	3 Radiology	4= 5-9
		2 Surgical	1= 10-14
		1 Rheumatology	1 =20+
Clinical Specialist	2	1 M.R.I	2 = 5-9
		2 C.T.	
Radiographer	6	4 Radiography	4 = 5-9
		2 Ultrasound	2 = 10-14
		1 C.T.	
		1 M.R.I	
		Participants report multiple specialties	
Radiation Safety Officer for Clinical Specialists	1	1 General	1 = 5-9

### Interview Design and Content

A review of the existing literature provided the groundwork for the semi-structured interviews. The interviews aim to explore the role of PACS in the workflow of each clinician, investigating their requirements for PACS, the perceived importance of sterility when interacting with PACS, as well as attitudes toward touchless interaction with PACS.

The study presented here forms part of a wider project that explores new technologies for user interaction with PACS, and aims to inform the development of future systems.

### Analysis

Interviews were audio-recorded and transcribed verbatim, with each participant assigned a unique ID code for anonymity. All data were analyzed thematically. Coding was performed using the approach described by Gale et al. [14]. Data for the interviews were analyzed independently by two researchers. Discussions were held to create themes and sub-themes, with the transcripts revisited until agreement was reached. The method of constant comparison was used, and key themes were identified and developed from the transcripts.

## Results

The findings are presented under the following headings: adoption and evolution of PACS, locations and roles, tasks and features, workflow, performance issues, training, and touchless interaction and sterility.

### Adoption and Evolution of PACS

HPs report that the introduction of PACS had a dramatic impact on the clinicians’ working day, bringing a newfound convenience to the clinical workflow. However, despite the fundamental nature of PACS in the clinical workflow, multiple HPs commented that they had observed no appreciable improvements over time. If anything, it is noted that PACS has become less usable over time for the typical user.

SREG4: “What stands out the most is that in 12 years, there’s been very little change.”

Though there are multiple providers of PACS solutions, HPs reported finding little difference between the various offerings.

Consultant Rheumatologist (CR1): “They all appear the same to me, to be honest.”

#### Impact on work practices

Participants are positive about the beneficial impact of PACS on their work practices. PC. 1: “I could easily see an X-ray and give my expertise based on an X-ray. That could only be done through PACS. It’s convenient. It allows you to outsource the expertise.”

Analytic functionality integrated with PACS is also mentioned, and these capabilities are likely to increase in the future [15]. PC 1: “I use engines of artificial intelligence to actually get a better diagnosis ...you can make it more precise, faster, cheaper, more efficient, etc.”

### Locations and Roles

HPs report three primary locations where they would use PACS: dedicated hospital workstations specific to the user (e.g., a radiologist’s office), at a shared hospital workstation/desktop (e.g., in the nurses’ office, on the ward, or in the clinic), and in the operating theatre.

Users report that gaining access to computers to use PACS can be a challenge on shared hardware as there is not enough hardware for the number of users. One user states that they use PACS (SHO 8) “Wherever I can get a computer.” In terms of computing power, many HPs report a lack of dedicated PACS hardware. Often, they access PACS software on general-purpose desktops that are frequently described as slow and not powerful enough for their needs.

**Table 2:** Frequency of PACS Usage by Participant

Uses per day	Number of participants
<1	3 (10%)
1-5	6 (19%)
6+	22 (71%)

### Tasks and Features

#### Imaging

HPs report basic navigation and management tasks as being common, such as searching for and opening scans; deleting images if they are not wanted was also referred to, though much less frequently. Deletion of unwanted or flawed imaging is reported as being performed in the radiology department, generally at the time the scan is being performed.

There is a strong emphasis on efficiency; HPs refer to a ‘red dot system’, where images in a sequence are marked with a red dot by the radiologist to identify images of significance, allowing faster identification of key images when reviewing the report. Use of dynamic features to play a sequence of images is also reported.

The lack of online access to scans performed in some hospitals is a clear source of frustration for certain HPs; when a scan is needed in that scenario the imaging and reporting need to be burned to disc, sent to the relevant hospital, and then uploaded into the local PACS. As patients often have differing identifying numbers between hospitals, difficulties are encountered when trying to determine if a patient has had previous scans.

One radiographer notes that sometimes image uploading can go wrong, with images ending up in incorrect locations. Radiographer 5: “Sometimes there’s multiple names or slight changes. Like, we’ll say, into the wrong folders”.

Despite the high level of functionality that PACS offers, it is reported that “for the most part [it’s] basic functions, basic functions”(PC 1). Many HPs report using very limited subsets of PACS features. HPs from the radiology department report using a wider range of PACS features.

Across the interview process it became clear that various feature sets are shared by HPs in the same role. Further, it is clear that the feature set someone would use can change if they change role, e.g., one HP reported previously using inverting a lot in a different role, but now not using the feature at all.

Measurement is one feature where HPs either report having no use for the feature, or having significant use for it. Not all measurement occurs on PACS itself, with some being performed directly on the scanner before the imaging is added to PACS. The need to perform a measurement can be situational, depending on the information a scan contains.

##### Use of the PACS suite in multidisciplinary team meetings

Several HPs referred to multidisciplinary meetings (MDTs), and reported that a more extensive range of tools is employed during those meetings. It is during the multidisciplinary meetings that various specialties come together to plan treatment for a patient [16]. Medical imaging is a key tool in the MDT that allows the clinicians to leverage their expertise, resulting in more informed diagnosis.

SHO 1: “So obviously PACS is a pivotal part of that meeting. It’s loaded up on a big screen and everybody is looking at it, things are being measured, we’re looking for lymph nodes, things like that. But it’s the radiologist that’s operating, we’re all just viewing.”

##### PACS as a tool to enhance imaging during post-processing

HPs report that they need to adjust the contrast of scans when the scan exposure is less than ideal, with a number of reasons being cited.

HPs note that human error, differences between the image displayed on the scanner versus the image that is saved to PACS, or even the patient’s physical size can impact on the exposure of a scan being not quite right. In these scenarios, adjusting the contrast allows the HP to better interpret the scan. Some HPs note that they perform this contrast adjustment directly on the scanner before sending the image to PACS.

Zooming is relatively commonly reported as being useful, especially for investigating subtle features such as certain fractures. Usage of inverting (flipping black to white, and white to black) is heavily task related, e.g., chest X-rays are frequently inverted, while other forms of imaging are not. Inverting is described as very helpful for appropriate uses.

#### Labelling and Annotating

Labelling and annotating are not commonly reported as being used. One HP notes that annotating can be used to record information that would otherwise be lost, such as the state of the patient during the scan, e.g., if the patient was in a state of expiration (breathed out). Such annotations allow HPs to understand and make allowances for potential shortcomings in the imaging.

Radiation Safety Officer: “might not be evident on the x-ray. You just write the patient was expiration.”

### Workflow

One HP reports that sometimes the emergency department would order scans but then not collect the scan. Every scan must be viewed by the person who ordered the scan, and must be recorded as having been viewed. If a scan is not viewed by the ordering clinician, additional workload is created for other members of staff in resolving this issue. This can arise due to issues with the structure of the organization or in errors entering the ordering clinician. SHO 9: “Sometimes the primary consultant is incorrect. So, scans that are ordered get attributed to the wrong department.”

Of note is the reported simplicity of the average clinician’s PACS workflow. Though PACS supports a wide range of functions, the vast majority of interactions will only use a small portion of those functions. This large feature set is reported as having led to difficulties using PACS, with HPs reporting a desire for simplification of the user interface.

SREG4: “We’re just trying to log in, find the patient, look at a report, look at an image. Having two dozen different options in not really useful for most of us.”

#### PACS is Often used in Busy Environments

HPs report that they frequently use PACS in busy environments with high levels of noise and human traffic. Further, it is reported that multiple people would be performing the same role on a given day, sharing the PACS between multiple HPs (with each HP having their own login). HPs note that rather than there being a lack of space, there is an abundance of people.

#### PACS as a Part of the Operating Theatre Workflow

In the context of the operating theatre, it is noted that there is a circulating scrub nurse who can be directed to use PACS. However, it is also noted that this is very inefficient and inconvenient as often the user being instructed may not be familiar with PACS and may not fully grasp the intent of the surgeon.

SHO 1: “It’s such a pain to try and direct a circulating scrub nurse on how to get to the exact point, scroll to the exact slice that you want.”

#### The Impact of Clinical Governance and Data Protection on HPs’ use of PACS

It is reported that there is a workflow/clinical governance balance that must be maintained. In order to protect patient information, PACS systems log the user out after a period of inactivity. Some HPs report this period as being as short as 5 minutes. All PACS logout automatically, including PACS in the operating theatre. HPs report that in the operating theatre the mouse needs to be ‘jiggled’ periodically by an unscrubbed staff member to keep the system awake. If the system goes to sleep it will log out, and then the correct login details will need to be entered and the system logged back in. In the context of the operating theater, it is noted that this involves an additional staff member knowing the clinicians log in details. After logging back in, the correct image needs to be navigated to and displayed correctly. HPs describe this as being a significant frustration.

SHO 8: “That’s really frustrating because we’ll have the imaging up, everything logged in and on the image that we want, and then as soon as the computer goes to sleep it logs out.”

Some HPs report that the PACS they have access to do not allow multiple simultaneous windows. Similarly, it is reported that switching patients, and between RIS and PACS for the same patient would completely close one application in order to open the other. This is deemed to be very frustrating for the HPs. The lack of system integration can have a significant impact on user efficiency as, in order to move between different functionalities, they must sacrifice their existing progress through the interface.

CS 1: “...you have their information up in the RIS and you’re like ‘oh God, did they have a scan?’, you have to go to click on the imaging part, and it closes that down completely. It doesn’t even minimise the window, it actually just closes it.”

#### PACS in the WHO Surgical Safety Checklist

In the context of the operating theatre, there is a specific WHO checklist that aims to decrease errors and adverse events [17]. It defines the workflow for multiple stages of a surgery, including ensuring that essential imaging is displayed, and thus PACS plays an important role in the checklist.

### Performance Issues and Their Impact on User Attitudes

While not universal, many HPs report various performance issues when using PACS. The number one complaint is speed, with many HPs reporting that PACS would run very slowly, with an adverse impact on their workflow, both from the delay, and from creating a reluctance to look at imaging.

SREG4: “Everyone should be looking at the images ...but we are discouraged from doing that because the system is so incredibly slow.”

### Perceived Shortcomings in Training in PACS

A significant majority of HPs report having never received any formal training in PACS, with most learning by observing their colleagues. Many HPs report a lack of understanding of PACS, saying that there are features of the software that they do not know how to use and therefore simply ignore.

Intern 3: “Pretty much all of my PACS knowledge is self-taught, or like is taught by someone else on the go. I feel like I could function more quickly and efficiently if I had had more formal training.”

One HP reports that though PACS training is available, most people do not take advantage of the training. It should be noted that many HPs are unaware of any available training.

SREG 2: “I think people don’t possibly take those opportunities up because they’re like ‘oh, I’ve used that before’ ...So I think that perhaps it’s not that there isn’t access to opportunity in training in PACS but more so that people don’t avail of them properly.”

When asked whether they feel that training would be beneficial, most HPs respond in the affirmative, though a small minority say they do not think that training would be beneficial, or would only be beneficial for more junior staff. Some HPs suggest that a basic level of training should be provided when first being introduced to a hospital’s PACS, with further, more advanced, training being provided some time after (between a month and a year). Some HPs suggest that short refresher courses would also be beneficial.

SHO3: “...you don’t really understand what you’re going to be doing, and then it would probably be good to train you in your first week and then maybe after a month to touch base with people again.”

It is reported that the scope of PACS software can make it difficult to learn in a single training session. HPs expressed a strong appetite for ongoing microlearning.

CR2: “But you know you bombarded at the beginning, it can do this this this and you’re only going to retain a very small amount of it. To be honest what you’re trying to do is the very basics, how do I call up the image and how do I report. But there are lots of other things you can do that most of us never use because you know you might be told on the first day but that’s no good. You really need to kind of to be retrained on it.”

It is suggested that PACS training could be incorporated as a component of other forms of training.

SHO3: “maybe as part of the surgical training or the medical training.”

Due to the fundamental nature of PACS, it is also suggested that PACS should be sufficiently intuitive as to not require face-to-face training. Instead, the majority of functions should be intuitive to use, with more advanced features available in additional views.

SREG4: “It shouldn’t really be required. So, a PACS system is so fundamental that training should either be able to be almost non-existent, then it’s so straightforward as you’re logging in for the first time, that your training is like a tutorial when you open up a new app on your phone.”

#### The Impact of Poor User Interface Design on User Understanding of the Software

Many HPs express a feeling that they lacked a proper understanding of PACS, opining that with a greater understanding they would be more efficient in using PACS, and would be able to leverage a greater number of tools to achieve more. This lack of understanding is attributed to a combination of an unfriendly user interface and a lack of training. HPs confirm that they would be more likely to use PACS if they feel more familiar with it.

HPs report that PACS is unclear and unintuitive to use, suffering from complex UI and poor UX. Even basic and essential features such as comparing images are described as being difficult to use, with this difficulty being attributed to a lack of understanding of the software. HPs report adopting manual solutions that lack the efficiency of built-in tools to get around their difficulties in using PACS.

### Touchless Interaction and Sterility

#### The Perceived Lack of Importance of Sterility Among PACS Users

A significant majority of HPs report that sterility is not important to them when using PACS. Most HPs state that this is because they would have an opportunity to wash their hands between using PACS and interacting with a patient. Those users with a dedicated workstations (such as radiologists) report that they are not concerned with cross-contamination. CR 2: “No, because essentially it’s my own grime...I don’t tend to use anyone else’s so I’m not worried about that at all.” A few HPs report that as they have no patient interactions, sterility is not a concern, though COVID-19 was cited as being a motivation for an increased awareness of the potential need for sterility.

SREG 6: “In the light of COVID it would be more important, particularly in a hospital that has seen a lot of COVID, and the terminals are almost never wiped down.”

HPs who use PACS in the operating theatre report that sterility is important to them in that context especially.

#### User Attitudes to the Potential of Touchless Interaction with PACS

Most HPs react positively to the idea of touchless interaction with PACS being available. In particular, HPs are easily able to visualize using voice-controlled shortcuts to speed up their workflow. HPs note that many tasks in PACS take a large number of clicks to achieve, with each click potentially taking a long time, and minimizing clicks is seen as an important requirement.

HPs also express positivity toward the idea of using a touchless system during aseptic procedures, or at times when the HPs hands might be busy. It is clear that, while touchless interaction might not be ideal for all environments in a hospital, there are a number of situations where the idea of access to touchless interaction of PACS is very appealing for HPs.

SHO 2: “I suppose if the technology was there it would be fine. You’d just need to make sure the theatres would just have to be set up properly so that there’s actually room for someone to move and make gestures, cause often theatres can be so tightly packed surrounding the computer that it would be hard to maintain sterility and perform gestures.”

Social context is also seen as affecting the acceptability of voice and gesture-based interfaces. PC 1: “In the operating room we also work with head gestures like nodding and things like that. It was just, you look almost stupid. So people would make fun of you.”

#### PACS and Touchless Interaction in the Operating Theatre

HPs report that PACS use in the operating theater is not a given, but rather is situation dependent. HPs note that when PACS is used in theatre it is very important. The operation is planned around a few key image(s), which are displayed from the start until the end of the operation. HPs in our sample reported that any interaction with PACS during a procedure is generally unplanned, with all imaging having been reviewed in advance of the operation.

SREG 1: “Typically you’d have a screen up in theatre and you’d have one key image up that you’re interested in. ...Then you can plan around it, just to refer back to just so you can know if your screws are in the correct orientation or something like that.”

HPs react favourably to the idea of touchless interaction with PACS in the operating theater. Currently, to directly interact with PACS the clinician needs to unscrub, interact with PACS, and then rescrub, which can be a time-consuming process. Alternatively, the clinician can direct another member of staff to interact with PACS on their behalf. SHO 3: “It would be really nice to be able to control the images yourself while you’re scrubbed in theatre wearing your gloves and your mask and not having to ask a nurse, a scrub nurse, ’scroll up, scroll down, go back, go this’.”

HPs report that they would be happy to adopt a robust touchless system, especially in a scrubbed environment, noting that voice dictation has already been successfully adopted in their hospitals.

#### The Preference of HPs for Voice Control

Many HPs express an interest in being able to use voice control to interact with PACS. Many people can visualize themselves leveraging voice commands to accelerate their workflow. Voice dictation is already commonplace in hospitals, so the use of voice as an interaction method is already commonplace. Gesture interaction by contrast is harder for people to visualize themselves using. Gesture-based interactions are not currently used in hospitals, resulting in a greater learning curve for gesture as compared to voice control.

SHO 11: “I don’t think there would be a downside...for me if you pull up simple commands, for example when comparing chest x-rays, if you have a command like ‘show me the [unclear] x-ray of the chest’, I mean that would be so useful rather than trying to trawl through all the investigations and find the last one.”

HPs report that they would be happy with some specific training for voice control if it improves performance. CS 1: “If you are trained to use specific words, or if you have to put it in chart number this and then it recognises that you’re searching for a patient.”

Based on feedback from HPs, there exists a desire for effective shortcuts within PACS. Many HPs express a desire to issue commands such as ‘display all images for patient X’. Aside from the convenience of being a verbal command, the greatest benefit of this instruction is that it reduces the complexity of performing the task, combining several actions into one.

Several HPs raised a potential issue regarding noise levels and the feasibility of voice control in the operating theatre. There can be many people talking at once, and in some scenarios music playing too. They assert that a voice control system would need to be very robust to background noise to be of use in a real-world environment. SHO 8: “It’s very noisy. And there’s always beeps and machines going, and a lot of people talking. So it would need to account for that....If we need to use it while we’re scrubbed, it tends to be, you know, an unplanned use.”

One other potential issue for voice control is data protection and privacy. While this issue is only raised by PC 1, with regulations such as GDPR protecting data privacy within the EU, any touchless technology needs to designed in such a way as to properly protect patient information.

PC 1: “Sometimes, for example, voice interface there can be a little privacy issues where you need to be aware of your surroundings. You may not want to give information that you could otherwise just type.”

#### Adoption of PACS Technologies

Overall, HPs express a positive attitude to adopting a touchless PACS. One HP asserts that new technologies in the hospital are often adopted from the bottom up, with junior doctors trying the technology first and word of the technology trickling up through the ranks until either the technology is adopted or fails to gain traction.

Intern 2: “You’ll have people starting to use it in the hospital and buy in from a couple of people, then that gets around...Generally it’ll be more junior doctors and if they find that it’s more efficient that’ll catch on ...If you had a negative experience the first time you might be less inclined to try it again, but if you had feedback from other people that it was very easy to use that would change your practice.”

A negative attitude is expressed by some HPs towards the adoption of touchless PACS, reporting that if there is not a clear benefit to their day to day work, then they would be unlikely to adopt the technology.

Some HPs note that they would like to have the option of both touchless interaction and conventional interaction methods, at least initially. It is noted that for seated interaction with PACS, touchless interactions such as gestures would be unlikely to be used due to the convenience of the mouse and keyboard. However, combining voice control and mouse and keyboard is suggested as being a beneficial combination as regards workflow efficiency.

#### The Central role of Radiology Reports in the Clinical Workflow

HPs in Ireland report the implementation of a nationwide system, National Integrated Medical Imaging System (NIMIS) that exists alongside PACS; NIMIS has come to replace a number of the functions of previous PACS setups, most notably the reporting element.

Although the content of the imaging is considered important, HPs note that often the report is of greater importance than the imaging, with some saying that they would only open the scans if they need to check something in the report. As the report is considered the authoritative interpretation of the scan, more junior HPs in particular report a preference for relying on the report rather than trying to interpret the scan themselves.

HPs report that all scans are officially interpreted by radiologists, with results being available to view generally by the next day. Many HPs report that, in the interest of speed, for more simple scans, such as chest X-rays, they would often make an initial assessment of the scan before the radiologist report is available. HPs note that often time is of the essence, and there is an emphasis placed on getting through as many patients as possible.

#### Integration of PACS with Other Systems

While there has been progress toward greater levels of integration between PACS and other EHR systems, HPs report that there are still significant shortcomings in this area. HPs note that this reduces their ability to carry skills between hospitals, often having to learn how to use unfamiliar software to perform familiar tasks.

SREG4: “Once you’re in the PACS, even you’re using an EHR, they tend not to be well integrated ...Even in private hospitals, which have relatively well integrated electronic records, the PACS tends not to work with it.”

HPs note that they tend to move between solutions based on the strengths of each system, e.g., between PACS and NIMIS.

## Discussion

### Summary of Results

Exploring the experiences and preferences of HPs when interacting with digital imaging, it is clear that for many HPs, PACS is an important part of their workflow, and there are significant issues with existing PACS. For many HPs there is a clear sense that they feel that they lack awareness of PACS features, and that additional training and improved usability would be of benefit. Usage of the various PACS features is aligned with a HP’s current role, with many users using only basic commands. HPs express general positivity to adoption of touchless interaction with PACS, with most user’s expressing a desire for voice control.

### Comparison with Existing Literature

In this section, we review our results in the context of existing studies on the use of PACS.

#### Tasks and features

The fact that multiple users can access images simultaneously once they are stored on PACS is raised in our interviews and is highlighted as a benefit by van de Wetering et al. [18].

Similar to the accounts in our interviews, Fridell et al. note the superior ability to display 3-dimensional reconstruction as an important feature enabled by the advent of new technology [19].

The HPs interviewed like to access the radiology report alongside the image, but report that it is not always easy in current systems. So too, Top’s results show that the majority of users consider the availability of radiology reports alongside imaging to be useful [20]. Top reports that reporting times decreased by 25% after the introduction of PACS [20]. Although this study did not formally investigate this question, the HPs at interview report greater efficiency following the implementation of PACS.

The importance of having a reliable experience while using PACS is raised in a recent study by Roseland et al (2019) [21] who report that a “stable system with predictable behaviour” that minimises “repetitive non-value-added work”, supports “interoperability” and with “near-instantaneous load times” are key requirements in any new PACS system.

#### Workflow

HPs commented on the lack of a consistent identifier for patients between hospitals, noting that it could make finding previous scans for a patient a challenge. In common with several countries, there is no national identity number program currently in place in the Irish healthcare system, even though legislation has been passed to enable this [22]. By contrast, many countries such as the Swedish healthcare system make use of a unique national identifier to identify patients. Such an electronic system’s primary purpose is to promote the medical care of individual patients, as well as allowing for their effective management, especially over time [23]. In addition to effective patient management, the unique national identifier also allows “medical data to be used for educational purposes, research and quality assurance schemes” [23]. Currently, it is not easy to compare two scans from a patient if the scans are performed in different institutions. Since these scans will have different identifiers, the system ‘sees’ these records as coming from different individuals, if the records are not already linked.

HPs in our study report that errors have occurred when storing images to PACS, with images being stored in incorrect locations. This error is recognized in the work of van de Wetering et al., who advises that it is vital to perform a check to verify that images have been correctly uploaded from PACS to the correct patient directory [18].

Echoing the desire for greater efficiency in the user interface in our study, Gale et al. write of the large number of “clicks on the mouse” required, which results in a loss of efficiency for the user [24]. HPs expressed a desire for a significantly simplified user interface, stating that the current complexity of PACS interfaces is a barrier.

#### Integration of PACS with Other Systems

The advantages of integration between PACS and other EHR systems are clear. According to Cohen et al., patient clinical information is not always available at point of care, instead being stored locally where it is created [25]. There are clear shortcomings to this situation in terms of patient care. “The current medical system needs to be integrated, secured, and available to health professionals and patients” [26]. A higher degree of integration can help overcome the shortcomings of standalone PACS solutions, such as local authentication, local access control, inconsistent patient identities, and local audit trail. Industry standards such as DICOM and HL7 were key to enabling integration of diverse data sources [25]. It is clear from our study that while progress toward integration has been made, further work is required on improving and standardizing the user experience.

#### Perceived shortcomings in training in PACS

Consistent with our study, Top reports that half of responders have no training to use PACS, with half of those responders reporting that no training had been offered [20].

Cox reports that users who receive formal training say they find that PACS workstations are easy to use, albeit sometimes “fiddly” [27]. This reinforces Top’s report that there can be significant differences between hospitals, potentially due to differences in types of PACS software used, or levels of training and experience [20].

Difficulties using PACS can lead to a requirement for external expertise; this is echoed in the work of Fridell et al., who say “If these technicians cannot solve the problem, the vendor’s technicians are called in. This makes the technology more distant from the radiographer, just as it makes the entire solution more complex than before.” [28].

*Adoption* Supporting the potential for voice noted in the study, Langer report that speech-recognition has a significant impact on productivity (up to 70%) for production of radiology reports, concluding that the adoption of PACS or speech recognition, or both improve report turnaround time [29]. This result is echoed by Lepanto et al., who reports significant decreases in dictation turnaround times one year after PACS implementation across several sites [30].

In contrast to earlier work, PACS is seen in our study as a stable and technology and integral part of the hospital workflow, but the introduction of new PACS software is seen as challenging and potentially disruptive. Thus, difficulties encountered in earlier PACS implementations [28] may arise again as new software is introduced. Paré and Trudel note that “merely deciding to adopt PACS does not guarantee success; effective PACS implementation is also necessary.” [31].

### Strengths and Limitations

We address a number of gaps in existing evidence by exploring HP experiences with current PACS installations, as well as their attitudes to touchless interaction with PACS. The qualitative approach taken provides insight into routine usage. The study explores the experiences of a range of stakeholders to provide an overview of the experiences and needs of health professionals using PACS in hospital environments.

The main limitation of the study is that participants were primarily recruited from Irish hospitals. As such, users in other countries may have different experiences with PACS, and different information systems used in conjunction with it. While the overall sample size is limited by the availability of clinicians, saturation was reached in the analysis of interviews, with later interviews confirming issues already identified in the analysis.

### Implications and recommendations

There is a clear appetite among HPs for significant improvements to existing PACS. While some of the changes needed fall outside the scope of this study, e.g., poor server performance or increased training, other improvements can be brought by consideration of how users interact with PACS. As workflows continue to evolve over time, it is of value to consider novel interactions with PACS, such as those enabled by touchless interaction technologies. The operating theatre is the location where would see the most obvious benefit of touchless interaction. Currently, it is arduous for clinicians to interact with PACS when scrubbed up. Either they must break sterility and re-scrub, or provide instruction to another staff member who may be less familiar with PACS. Touchless interaction, whether voice control or gesture interaction, would allow HPs direct control of PACS and would provide reassurance to surgeons in the operating theatre. It is clear that the hospital environment poses a number of technical challenges for any touchless interaction system. The hospital is a loud, busy environment where both voice control and gesture-based recognition will experience challenges. It is also an environment where efficiency is a requirement. In order for a technology to be successfully adopted by HPs, it must deliver a high-quality experience and must avoid slowing HPs down in their day-to-day tasks.

For PACS developers, there are a number of key points. Firstly, though PACS provides a powerful set of features, it suffers from poor usability. PACS interfaces are too complex for the average user to effectively exploit the set of features most useful to them. The study suggests that improvements to usability would enable more effective use of PACS. For example, users should be able to view a simplified interface that surfaces the tools relevant to their role.

Secondly, PACS would benefit from additional interaction mechanisms, especially voice commands. There is a strong appetite among clinicians for voice commands that could speed up their workflow. For example, the ability to verbally instruct a PACS to display all images for a patient, including previous imaging, is mentioned by a number of clinicians as a functionality they would highly value.

## Future Work

This work has raised a number of questions that would be best addressed by future work. There is a clear need for improvements to PACS training for clinicians, and more convenient support could also be helpful. Future work investigating learning delivery methods would be of benefit. Research should focus on determining which learning tools are most effective and best suit the clinician’s workflow. In order to best target training, there would be great benefit to research into which PACS tools are most used by various user groups. This would allow training be less overwhelming, enabling the clinician take greater advantage of appropriate subsets of PACS features. PACS access from mobile devices, such as smartphones and tablets, could provide a powerful new tool to the clinician’s arsenal. This would be of particular interest in the context of non-radiologist users, whose needs may focus more on accessing reports with images to provide additional context.
